# Long-Term Treatment with Low Doses of Methamphetamine Promotes Neuronal Differentiation and Strengthens Long-Term Potentiation of Glutamatergic Synapses onto Dentate Granule Neurons

**DOI:** 10.1523/ENEURO.0141-16.2016

**Published:** 2016-07-11

**Authors:** Sofia Baptista, Joana Lourenço, Nuno Milhazes, Fernanda Borges, Ana Paula Silva, Alberto Bacci

**Affiliations:** 1 Institute of Pharmacology and Experimental Therapeutics, Faculty of Medicine, University of Coimbra, 3000-548 Coimbra, Portugal; 2 Institute for Biomedical Imaging and Life Sciences, Faculty of Medicine, University of Coimbra, 3000-548 Coimbra, Portugal; 3 Sorbonne Universités, Université Pierre et Marie Curie, Unité Mixte de Recherche (UMR) S 1127, Institut National de la Santé et de la Recherche Médicale U 1127, Centre National de la Recherche Scientifique UMR 7225, and Institut du Cerveau et de la Moelle épinière, 75013 Paris, France; 4 Institute of Health Sciences-North, 4585-116 Gandra, Portugal; 5 Department of Chemistry and Biochemistry, Faculty of Sciences, Centro de Investigação em Química da Universidade do Porto, 4169-007 Porto, Portugal

**Keywords:** ADHD, dentate gyrus, methamphetamine, neurogenesis, synaptic plasticity

## Abstract

Methamphetamine (METH) is a psychostimulant, affecting hippocampal function with disparate cognitive effects, which depends on the dose and time of administration, ranging from improvement to impairment of memory. Importantly, in the United States, METH is approved for the treatment of attention deficit hyperactivity disorder. Modifications of long-term plasticity of synapses originating from the entorhinal cortex onto dentate granule cells (DGCs) have been proposed to underlie cognitive alterations similar to those seen in METH users. However, the effects of METH on synaptic plasticity of the dentate gyrus are unknown. Here, we investigated the impact of long-term administration of METH (2 mg/kg/d) on neurogenesis and synaptic plasticity of immature and mature DGCs of juvenile mice. We used a mouse model of neurogenesis (the G42 line of GAD67-GFP), in which GFP is expressed by differentiating young DGCs. METH treatment enhanced the differentiation of GFP^+^ cells, as it increased the fraction of GFP^+^ cells expressing the neuronal marker NeuN, and decreased the amount of immature DGCs coexpressing doublecortin. Interestingly, METH did not change the magnitude of long-term potentiation (LTP) in more immature neurons, but facilitated LTP induction in more differentiated GFP^+^ and strengthened plasticity in mature GFP^−^ DGCs. The METH-induced facilitation of LTP in GFP^+^ neurons was accompanied with spine enlargement. Our results reveal a specific action of long-term use of METH in the long-term plasticity of excitatory synapses onto differentiating DGCs and might have important implications toward the understanding of the synaptic basis of METH-induced cognitive alterations.

## Significance Statement

Methamphetamine (METH) abuse/misuse can induce memory deficits, but controlled drug prescription is approved by the Food and Drug Administration for the treatment of attention deficit hyperactivity disorder. Additionally, dentate gyrus (DG) neurogenesis contributes to the formation and consolidation of new memories. We therefore studied the effects of 7 d exposure to a low dose of METH in DG neurogenesis as well as its impact in synaptic plasticity. We observed that METH accelerated immature neuron differentiation and facilitates LTP in more differentiated immature neurons and mature DGCs. These effects might be the synaptic correlate of some of the METH-induced memory alterations.

## Introduction

Methamphetamine (METH) is a psychostimulant that is commonly known as a highly addictive drug of abuse, having a negative impact on the CNS (Gonçalves et al., 2014). Indeed, many studies have indicated that METH abuse induces deficits in working memory (Simon et al., 2000) and decreases hippocampal volume, which can be correlated with poorer word recall test results (Thompson et al., 2004). In rodents, hippocampal-dependent memory performance may depend on the dose of METH administered. Indeed, a high dose (30 mg/kg, i.p.) can induce memory deficits (Simões et al., 2007; Gonçalves et al., 2012), whereas a lower dose (1 mg/kg, i.p.) can improve spatial memory consolidation (Cao et al., 2013). Importantly, METH is approved by the U.S. Food and Drug Administration for the treatment of attention deficit hyperactivity disorders (ADHDs), under the commercial name Desoxyn, with an effective dose of 20-25 mg.

Adult neurogenesis occurs throughout life in the subgranular zone of the dentate gyrus (DG) and the subventricular zone (Lledo et al., 2006), and there is a strong link between hippocampal neurogenesis and memory performance, as immature DG neurons are preferentially activated and recruited into spatial memory networks (Kee et al., 2007; Garthe et al., 2009). Indeed, DG plasticity can be induced by stimulating the perforant path of the molecular layer, by tetanic, patterned, high-frequency or theta-burst stimulations (TBSs) that can trigger long-term potentiation (LTP) onto dentate granule cells (DGCs; Bliss and Lømo, 1973; Richter-Levin et al., 1994; Panja et al., 2014). It is widely believed that a long-lasting change in synaptic function is the cellular basis of learning and memory (Malinow and Malenka, 2002), and the most characterized examples of synaptic plasticity are LTP or long-term depression of glutamatergic neurotransmission in the CA1 and DG areas of the hippocampus. Likewise, recent evidence causally linked LTP with learning and memory in several brain areas, including the DG (Zeng et al., 2001; Whitlock et al., 2006; Nabavi et al., 2013, 2014; Gruart et al., 2015; Ryan et al., 2015). It is known that medial perforant path (MPP)–DG synapses are crucial for spatial learning, context-dependent learning, and episodic memory (Hargreaves et al., 2005). Indeed, MPP, as opposed to lateral perforant path, is strongly involved in spatial information processing (Hargreaves et al., 2005).

Some evidence indicates that neurogenesis can be affected by METH, depending on the dose and frequency of exposure. Indeed, self-administration with intermittent access to METH, which mimics a human recreational use, increases DGC proliferation and differentiation (Mandyam et al., 2008). Conversely, both short- and long-term use (daily access) decrease proliferation and differentiation followed by a reduced number of DGCs (Mandyam et al., 2008). *In vitro* studies also showed that METH induces cell cycle arrest in DG stem cells and impairs their self-renewal, resulting in their differentiation into the neuronal phenotype (Baptista et al., 2014). Also, under differentiation conditions, a nontoxic dose of METH can impair the maturation of DG stem/progenitor cells (Baptista et al., 2012). In this scenario, it is crucial to understand whether low doses of METH affect the functional properties of immature and mature DG cells. In the present work, we explored the effects of long-term (7 d) METH administration at low doses (2 mg/kg) on DG neurogenesis, and its influence on the synaptic plasticity of both immature and mature neurons. It is noteworthy that the METH injection protocol used in the present work mimics ADHD treatment. We found that METH accelerated the maturation of immature neurons, and strengthened LTP at more mature differentiating stages and fully developed DGCs. Our results indicate that long-term administration of low doses of METH strengthens synaptic plasticity at a specific maturation stage of DGCs and could therefore affect memory performance.

## Materials and Methods

### Mice

Experimental procedures followed national and European (2010/63/EU) guidelines, and have been approved by AP Silva institutional review boards. All efforts were made to minimize suffering and reduce the number of animals used in the procedures. Experiments were performed on G42 (GAD1-EGFP) mice, which were obtained from The Jackson Laboratory (Jax mouse line, Tg(Gad1-EGFP)G42Zjh; mouse strain datasheet #007677). In this mouse line, GFP^+^ cells are parvalbumin-expressing, fast-spiking interneurons in the neocortex (Chattopadhyaya et al., 2004) and immature DGCs of the hippocampus with a transient GABAergic phenotype (Cabezas et al., 2012, 2013). G42 mice were continuously backcrossed on a C57BL/6 background and were weaned at postnatal day 21 (P21), and only males were used for the experiments. Although hydrocephaly has been described in this mouse strain (https://www.jax.org/strain/007677), all hydrocephalus animals were discarded from experiments.

### METH injection protocol

METH was synthesized at the Organic Chemistry Department, Faculty of Pharmacy, University of Porto, Porto, Portugal, and was dissolved in 0.9% NaCl at a concentration of 0.8 mg/ml. P20–P24 G42 mice were injected with 2 mg/kg METH, or saline solution (intraperitoneally), every day for 7 d. Taking into consideration the duality of METH use (drug of abuse vs ADHD treatment), the protocol used here aimed at mimicking METH administration for ADHD treatment (the usual effective dose is 20–25 mg daily), in contrast to the already well known impact of METH abuse. Immunohistochemical, morphological, and electrophysiological experiments were performed 24 h after the last injection.

### Immunohistochemistry

Mice were anesthetized with ketamine (80 mg/kg, i.p.; Imalgène) and xylazine (20 mg/kg, i.p.; Rompun, Bayer), transcardially perfused with 4% paraformaldehyde (PFA), and cryopreserved in 30% sucrose. Brains were horizontally cut in 40-µm-thick slices in a cryotome (Microm HM450). Slices were rinsed with PBS and blocked in 10% BSA and 0.3% Triton for 2 h at room temperature. Slices were incubated with the following primary antibodies overnight at 4°C: chicken anti-green fluorescent protein (1:1000; Millipore); rabbit anti-doublecortin (DCX; 1:2000; Cell Signaling Technology); and mouse anti-NeuN (1:250; Millipore). Slices were incubated with the respective secondary antibodies for 3.5 h at room temperature, as follows: donkey anti-chicken IgG Cy2 (1:500); donkey anti-rabbit IgG Cy3 (1:600); and Alexa Fluor 647 donkey anti-mouse (1:400; all from Jackson ImmunoResearch). Slices were mounted in Fluoromount-G (Southern Biotech; Cabezas et al., 2013). Images were acquired on an inverted confocal microscope (SP2, Leica) and were obtained from stacks of 10–17 sections, 2.5 µm apart, using a 63×/1.32 numerical aperture objective. Images were acquired from four slices per animal of five saline- or METH-treated mice. Cell counts were calculated using ImageJ software. Results are expressed as the percentage of total GFP cells expressing DCX and/or NeuN.

### *In vitro* slice preparation and electrophysiology

G42 mice aged P28–P32 were anesthetized with isoflurane (Sigma-Aldrich) and were immediately decapitated. Brains were quickly removed into ice-cold cutting solution containing the following (in mm): 248 sucrose, 26 NaHCO_3_, 1 KCl, 1 CaCl_2_, 5 MgCl_2_, and 10 glucose (all from Sigma-Aldrich). The 350-µm-thick horizontal slices were then obtained using a vibratome (VT 1200 S, Leica) and transferred to an incubating chamber containing artificial CSF solution (ACSF) as follows (in mm): 126 NaCl, 2.5 KCl, 1.25 NaH_2_PO_4_, 26 NaHCO_3_, 2 CaCl_2_, 1 MgCl_2_, and 16 glucose (all from Sigma-Aldrich), bubbled with 95% O_2_/5% CO_2_ at 34°C for 30 min, followed by incubation at room temperature for at least 1 h before recording. Slices were then transferred to a submerged recording chamber, and neurons were visualized using infrared videomicroscopy in a microscope equipped with epifluorescence. Recordings were performed at 32°C in the continuous presence of the GABA_A_ receptor (GABA_A_R) antagonist gabazine (10 µm). Microelectrodes with 3-4 MΩ tip resistance were pulled from borosilicate glass capillaries [World Precision Instruments (WPI)] using a Flaming/Brown Micropipette puller P-97 (Sutter Instruments). Intracellular solution consisted of the following (in mm): 130 potassium gluconate, 10 KCl, 10 HEPES, 2 MgCl_2_, 5 phosphocreatine, 4 MgATP, 0.3 NaGTP, and 0.2 EGTA (all from Sigma-Aldrich), pH 7.3, with an osmolarity of ∼290 mOsm. DGCs were whole cell, patch clamped, and were recorded both in current- and voltage-clamp modes. Signals were amplified with a MultiClamp 700B patch-clamp amplifier, sampled at 10 kHz, and filtered at 4 kHz (Molecular Devices). Data were analyzed using the PClamp 10 software package (Molecular Devices) and GraphPad Prism software. GFP^+^ and GFP^−^ cells were characterized in current-clamp mode. Action potential (AP) firing was evoked by injecting 1-s-long depolarizing steps of increasing amplitude, starting at −25 pA and with increments of 10 pA. For LTP experiments, DGCs were recorded in voltage-clamp mode at a holding potential of −70 mV. A bipolar stimulating electrode, fabricated from a theta capillary (WPI), was filled with ACSF and placed in the middle third of the molecular layer in order to orthodromically stimulate the MPP. EPSCs were evoked by short (0.2 ms) pulses at 5 V using an isolation unit (Isoflex, A.M.P.I), triggered by the digital output of the digitizer (Digidata 1440, Molecular Devices) and controlled by PClamp version 10. LTP was induced by applying a TBS paradigm consisting of five bursts of five extracellular stimulations at 100 Hz, repeated at 5 Hz, and paired with postsynaptic depolarization at −30 mV. These bursts were delivered 16 times at 0.1 Hz (Bowden et al., 2012). Series resistance was continuously monitored, and recordings were discarded if it was >30 MΩ or changed >20%.

### Morphological analysis

Cells were filled with neurobiotin (3 mg/ml; Vector Laboratories) added to the intracellular solution. After fixation with 4% PFA followed by PBS rinses, cells were permeabilized in 2% Triton in PBS for 1 h and then incubated for 2 h with ABC reagent (avidin and biotinylated horseradish peroxidase complex; Vector Laboratories). Slices were rinsed in PBS (2× 10 min, 1× 15 min and 1× 1 h) and reacted with 3,3'-diaminobenzidine (Vector Laboratories) until the stain was visualized. Slices were mounted in 85% glycerol (Sigma-Aldrich) in PBS. Neurons were reconstructed using the Neurolucida software (Microbrightfield) using a 40× objective. Differential interference contrast images were acquired with a macroscope (AZ100, Nikon) from stacks of 11 sections, 2 µm apart, using a 5× objective. Neurobiotin-filled neurons were also visualized by fluorescence upon streptavidin aminimethylcoumarin (Jackson ImmunoResearch) staining, and images were acquired by confocal microscopy, as mentioned above. Spine density and area were determined using ImageJ. Spine density was assessed by counting the number of spines divided by the length of a dendritic segment. Spine areas were determined by drawing individual regions of interest (ROIs) outlining spine heads and necks, and the area of each ROI was then measured.

### Data analysis

Statistical significance was assessed using one-way ANOVA followed by Kruskal–Wallis test and by Dunn’s multiple comparison test or Mann–Whitney *post hoc* test, as indicated in the ﬁgure legends. Data are expressed as the mean ± SEM, and the statistical signiﬁcance level was set for *p* < 0.05. Cumulative distributions were compared using the Kolmogorov–Smirnov test.

## Results

### Methamphetamine accelerates immature dentate granule cell maturation

**Figure 1.**
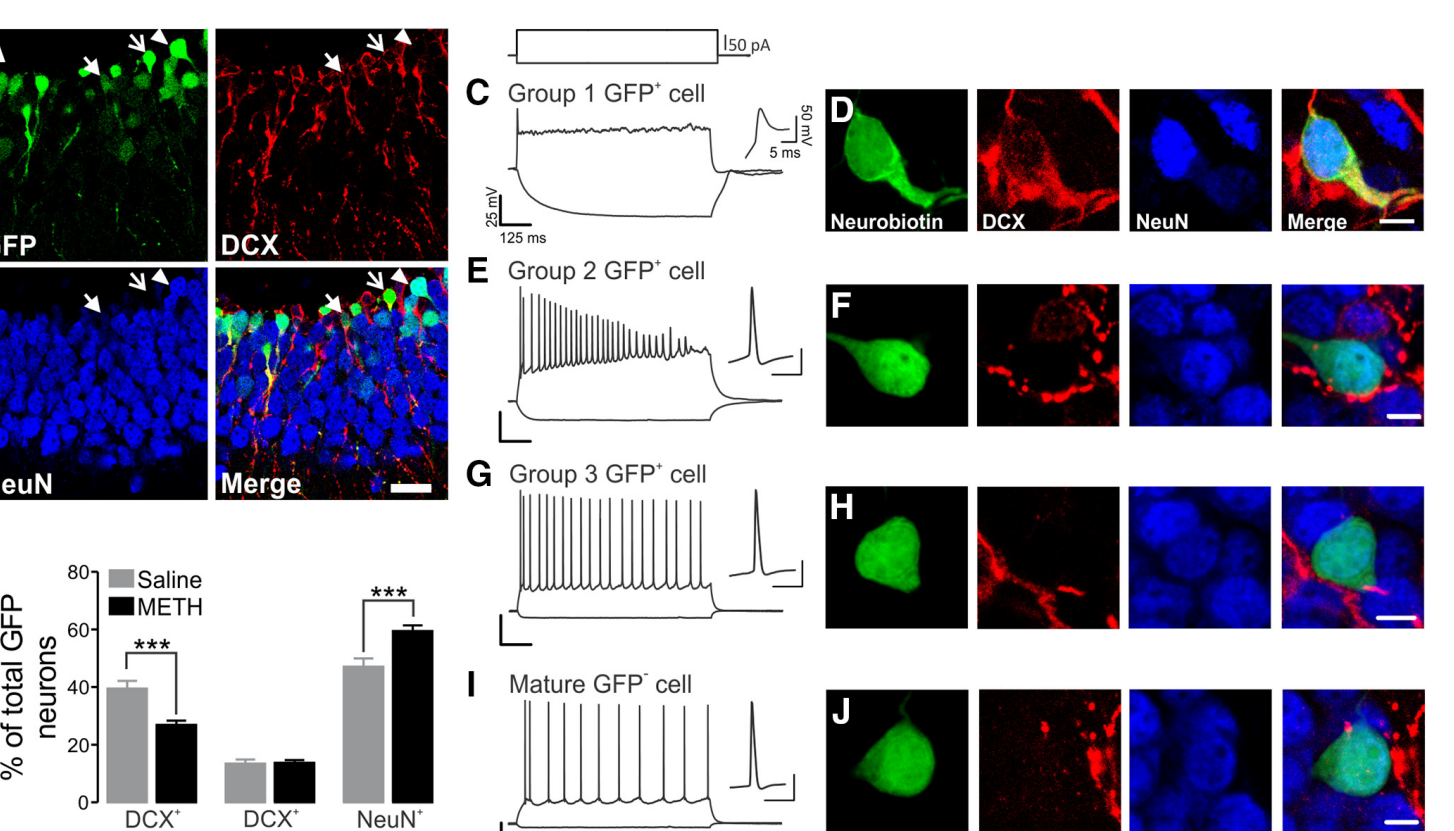
METH enhances the differentiation of immature DG neurons. ***A***, Representative confocal images exhibiting the different phenotypes of immature GFP^+^ DGCs. Open arrows, GFP^+^/DCX^+^ cells; closed arrows, GFP^+^/DCX^+^/NeuN^+^; arrowheads, GFP^+^/NeuN^+^. Scale bar, 50 µm. ***B***, Bar graph illustrating the effect of METH in GFP^+^ cell phenotype. Data are expressed as a percentage of GFP^+^ cells ± SEM from four horizontal slices/animal of at least five animals per condition. ***p < 0.001, significantly different from saline using Mann–Whitney *post hoc* test. ***C–I***, Current-clamp traces showing that GFP^+^ cells exhibit different AP firing profile patterns identified as group 1, 2, 3, and mature cells (***C***, ***E***, ***G***, ***I***), respectively, and the respective phenotype characterization (***D***, ***F***, ***H***, and ***J***). Cells were analyzed from at least three mice. Scale bar, 5 µm.

G42 mice were used as a model of DG neurogenesis, because GFP expression is transient and specific for immature DGCs (Cabezas et al., 2013). We performed immunohistochemical staining of 28–32-d-old mice, consistent with previous reports (Cabezas et al., 2012, 2013), and we found that GFP^+^ neurons corresponded to immature DGCs at different stages of neuronal maturation. DGCs expressed DCX alone (Fig. 1*A*, open arrows), coexpressed DCX and NeuN (Fig. 1*A*, filled arrows), or expressed NeuN (Fig. 1*A*, arrowheads). Long-term administration of METH (2 mg/kg/d, i.p., for 7 d) decreased the number of GFP^+^ cells expressing only DCX (saline, 39.42 ± 2.75% of total GFP cells; METH, 26.85 ± 1.54% of total GFP cells; *p* < 0.001) and increased the number of GFP^+^ cells that were positive for NeuN (saline, 47.12 + 2.84% of total GFP cells; METH, 59.42 ± 1.98% of total GFP cells; *p* < 0.001; Fig. 1*B*). METH treatment did not interfere with GFP^+^ DGCs coexpressing DCX and NeuN (saline, 13.46 ± 1.43% of total GFP cells; METH, 13.72 ± 0.95% of total GFP cells; Fig. 1*B*). Additionally, METH decreased the total number of GFP^+^-expressing DCX cells, regardless of whether they also expressed NeuN (saline, 53.27 ± 3.07% of total GFP; METH, 40.65 ± 2.08% of total GFP; *p* < 0.05). In contrast, it increased the total number of GFP^+^ cells expressing NeuN, regardless of whether they also expressed DCX (saline, 60.28 ± 2.97% of total GFP; METH, 73.09 ± 1.62% of total GFP; *p* < 0.05). Importantly, METH did not change the total number of GFP^+^ cells in each horizontal section of the DG (density of GFP^+^ DGCs: saline, 1.09 ± 0.35% cells/mm^2^; METH, 1.53 ± 0.47% cells/mm^2^; *p* > 0.05; *n* = 4 mice per group).

We then performed whole-cell current-clamp recordings from several GFP^+^ (*n* = 115 cells) and GFP^−^ (*n* = 69 cells) DGCs, and we found three major different firing patterns in response to depolarizing current pulses (Fig. 1*C*,*E*,*G*,*I*). Group 1 neurons fired only single, small-amplitude APs, irrespective of the intensity of stimulation (Fig. 1*C*); group 2 neurons generated few APs during the depolarizing current step, with a diminishing AP amplitude (Fig. 1*E*); group 3 neurons exhibit a fully blown, repetitive AP firing pattern (Fig. 1*G*), more similar to mature GFP^−^ neurons (Fig. 1*I*). Importantly, GFP^+^ DG neurons belonging to groups 1–3 displayed significantly different values for passive membrane properties and action potential waveforms (Table 1). This was consistent with previously described functional changes of developing DG neurons (Mongiat et al., 2009; Cabezas et al., 2013).

**Table 1.** Action potential waveform and membrane properties values of GFP^+^ DG neurons

	Group 1		Group 2		Group 3	
	Saline	METH	Saline	METH	Saline	METH
AP peak (mV)*	−0.97 ± 1.70	−0.72 ± 4.28	34.59 ± 2.26	40.95 ± 1.70	45.27 ± 1.72	46.18 ± 2.50
AP amplitude (mV)*	29.38 ± 3.10	30.25 ± 5.99	80.57 ± 3.86	85.83 ± 2.81	95.24 ± 1.47	94.43 ± 2.94
AP area (mV · ms)	78.71 ± 3.63	75.44 ± 6.68	91.27 ± 5.42	84.50 ± 7.36	92.91 ± 2.97	85.47 ± 3.84
Half-width (ms)*	3.38 ± 0.45	3.35 ± 0.69	0.98 ± 0.10	0.79 ± 0.06	0.85 ± 0.04	0.79 ± 0.05
C_m_ (pF)*	21.66 ± 0.77	19.96 ± 1.18	40.02 ± 3.21	42.39 ± 7.32	56.00 ± 3.13	49.96 ± 3.22
R_in_ (GΩ)*	2.32 ± 0.16	2.35 ± 0.18	0.68 ± 0.11	0.92 ± 0.15	0.38 ± 0.03	0.36 ± 0.03
RMP (mV)*	−38.00 ± 1.76	−36.25 ± 4.09	−62.01 ± 3.39	−58.79 ± 2.87	−68.17 ± 1.20	−69.21 ± 1.53

Values are given as the mean ± SEM. METH induced no effect in all parameters analyzed. C_m_, membrane capacitance; R_in_, input resistance; RMP, resting membrane potential.

*Significantly different, using Kruskal–Wallis test followed by Dunn’s *post hoc* test or Bonferroni’s *post hoc* test. Asterisks correspond to statistical significance (*p* < 0.05) between group 1 and group 2 GFP^+^ DGCs, and between group 2 and group 3 GFP^+^ DGCs in saline conditions.

DGCs were regularly filled with neurobiotin during electrophysiological experiments, and then immunolabeled with DCX and NeuN. Group 1 GFP^+^ cells expressed both DCX and NeuN (Fig. 1*D*), whereas both group 2 and 3 GFP^+^ cells expressed NeuN alone (Fig. 1*F*,*H*). As expected, mature DGCs expressed NeuN (Fig. 1*J*). Overall, Group 1, 2, and 3 DGCs corresponded to 27%, 24%, and 49% of our total recorded cells (*n* = 74). Although METH treatment increased the number of NeuN-expressing DGCs, it did not change the incidence of finding group 1 (25%), 2 (31%), or 3 (44%) cells in our recordings (*n* = 76; data not shown; *p* > 0.05). Importantly, METH treatment did not alter DGC excitability, measured as an AP waveform, and passive membrane properties in all groups (data not shown). Surprisingly, we could not find GFP^+^ neurons expressing only DCX in these recordings, possibly due to undersampling during our electrophysiological recordings associated with immunohistochemistry characterization (*n* = 36 cells). It is likely that group 1 neurons encompass neurons expressing either DCX only or DCX/NeuN, and that they could be located nearby the subgranular zone. Indeed, DGCs expressing only DCX showed firing properties similar to our group 1 neurons (Lledo et al., 2006).

Overall, we found that GFP^+^ cells in juvenile G42 mice correspond to differentiating newborn neurons at different stages of maturation and that METH treatment promoted DGC differentiation.

### Methamphetamine effects on synaptic plasticity in different DGCs groups

Although some studies have pointed out that METH induces cognitive deficits (Simon et al., 2000), others showed that METH can improve memory performance (Cao et al., 2013; Silber et al., 2006). Also, the integration of immature neurons into pre-existent DG circuitry is known to have a prominent role in memory processes (Kee et al., 2007; Garthe et al., 2009). We therefore aimed at clarifying whether long-term treatment with a daily low dose of METH affects the synaptic plasticity of both immature and mature DG neurons.

**Figure 2.**
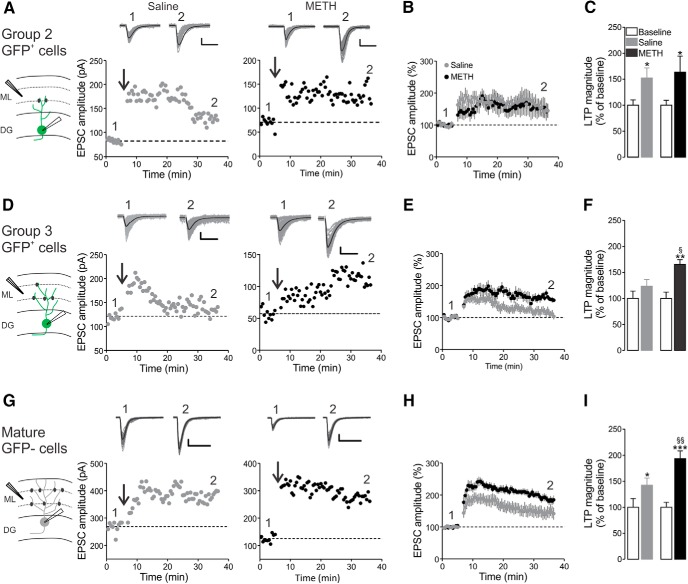
METH effects on LTP in differentiating GFP^+^ neurons and in mature DGCs. ***A***, The diagram on the left illustrates the recording configuration. Middle, Representative time courses of EPSCs in both saline-injected (open symbols) and METH-injected (filled symbols) mice of group 2 GFP^+^ cells. Insets, Representative 60 traces (gray lines) and their average (black lines) before (1) and ∼40 min after (2) TBS (arrow). ***B***, Population time course of group 2 DGC plasticity. ***C***, LTP magnitude assessed after ∼40 min (2) normalized to baseline (1) in group 2 DG neurons. ***D–F***, As in ***A–C***, but for group 3 cells. ***G–I***, as in ***A–C*** and ***D–F***, but for mature, GFP^−^ DGCs. Data are expressed as the mean ± SEM. *p < 0.05, **p < 0.01, and ***p < 0.001, significantly different from baseline using the Mann–Whitney *post hoc* test. §*p* < 0.05, §§*p* < 0.01, significantly different from saline, using the Mann–Whitney *post hoc* test.

GFP^+^ and GFP^−^ neurons were recorded in voltage clamp, in the continuous presence of the GABA_A_R antagonist gabazine (10 µm), and excitatory postsynaptic currents (EPSCs) were evoked by extracellular stimulation of the MPP. The stimulation of MPP was confirmed as EPSCs were strongly reduced after application of the selective mGlu_2_ receptor agonist DCG-IV, which was shown to be selectively expressed by MPP terminals (Chiu and Castillo, 2008; data not shown). Group 1 neurons showed few or no EPSCs (data not shown) and therefore could not be studied further. Group 2 cells were able to generate evoked EPSCs and showed robust LTP after TBS application (Fig. 2*A*). Indeed, the increase in synaptic strength was verified in both saline (baseline, 82.22 ± 1.46 pA; last 5 min after TBS, 128.30 ± 3.01 pA; *p* < 0.001, paired *t* test; *n* = 7) and METH (baseline, 70.21 ± 3.22 pA; last 5 min after TBS, 128.70 ± 6.27 pA; *p* < 0.001, paired *t* test; *n* = 8; Fig. 2*A*). Both in saline and METH, EPSC amplitude increased when compared with the respective baseline values (Fig. 2*B*), but METH did not induce any effect on the magnitude of LTP in group 2 GFP^+^ cells (*p* > 0.05; Fig. 2*C*).

Surprisingly, group 3 cells consistently exhibited only a transient increase in EPSCs in response to TBS in saline-treated animals (Fig. 2*D–F*). This could not be considered as LTP, because it was not sustained for >20 min (baseline, 96.21 ± 11.39 pA; last 5 min after TBS, 109.80 ± 17.25 pA; paired *t* test; *n* = 12 cells; Fig. 2*D*). Interestingly, METH treatment promoted the expression of a long-lasting LTP in group 3 DGCs (EPSCs at baseline, 64.80 ± 5.69 pA; EPSCs in the last 5 min after TBS, 105.20 ± 9.42 pA; *p* < 0.001, paired *t* test; *n* = 7 cells), which were significantly different from those in the saline group (*p* < 0.05; Fig. 2*E*,*F*). Finally, in mature neurons (not expressing GFP), TBS application increased EPSC amplitude in both the saline group (baseline, 138.90 ± 18.44 pA; last 5 min after TBS, 191.10 ± 28.50 pA; *p* < 0.05, paired *t* test; *n* = 12 cells; Fig. 2*G*) and the METH group (baseline, 128.20 ± 15.75 pA; last 5 min after TBS, 234.90 ± 23.35 pA; *p* < 0.001, paired *t* test; *n* = 10; Fig. 2*G*). Remarkably, however, METH strengthened LTP significantly compared with saline (*p* < 0.05; Fig. 2*H*,*I*).

Overall, long-term METH treatment did not interfere with synaptic plasticity in more immature DGCs (group 2 GFP^+^ neurons), but it promoted and enhanced LTP at more differentiated stages (group 3 GFP^+^ neurons) and in fully developed GFP^−^ neurons.

### Methamphetamine treatment did not change DGC dendritic morphology

**Figure 3.**
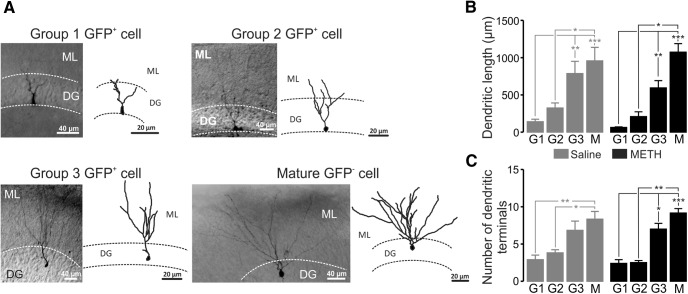
DG immature neurons exhibit different morphologies. ***A***, Example micrographs of neurobiotin-filled DGCs of all groups (left) and their digital reconstructions (right). ***B***, ***C***, Bar graphs representing the total dendritic length (***B***) and the number of dendritic terminals (***C***) of GFP^+^ and mature DGCs, showing no effect of METH. Data are expressed as the mean ± SEM from at least four mice. *p < 0.05, **p < 0.01, and ***p < 0.001, significantly different using the Kruskal–Wallis test followed by Dunn’s *post hoc* test. G1, Group 1 GFP^+^ cells; G2, group 2 GFP^+^ cells; G3, group 3 GFP^+^ cells; M, mature DGCs.

The METH-induced effects on synaptic plasticity in DGCs could be accompanied by morphological changes of DGCs. We then tested whether long-term exposure to METH induced dendritic alterations at different differentiation stages. We filled GFP^+^ neurons from the three groups with neurobiotin and analyzed their dendritic morphology. Group 1 cells showed few and short ramifications, and cell bodies were mostly confined in the inner dentate granule layer (Fig. 3*A*). Complexity was more prominent as GFP^+^ DGCs belonged to groups 2 and 3, which gradually acquired the typical morphology of mature DGCs (Fig. 3*A*). These changes were statistically significant across different DG groups in both saline- and METH-treated animals. Overall, METH administration did not produce any effect on dendritic length (group 1: 63.62 ± 7.363 vs 140.40 ± 33.43 µm, *n* = 5 vs n = 10; group 2: 208.40 ± 65.26 vs 323.10 ± 69.17 µm, *n* = 5 vs *n* = 11; group 3: 592.80 ± 98.90 vs 786.20 ± 164.60 µm, *n* = 10 vs *n* = 6); mature: 1073.00 ± 115.60 vs 956.60 ± 181.60 µm, *n* = 13 vs *n* = 9; METH vs saline, respectively, *p* > 0.05 in all cases; Fig. 3*B*). Similarly, METH did not change the number of dendritic terminals (group 1: 2.40 ± 0.51 vs 2.90 ± 0.62, *n* = 5 vs *n* = 10; group 2: 2.50 ± 0.29 vs 3.82 ± 0.42, *n* = 5 vs *n* = 11; group 3: 7.00 ± 0.77 vs 6.83 ± 1.25, *n* = 10 vs *n* = 6; mature: 9.15 ± 0.60 vs 8.33 ± 1.04, *n* = 13 vs *n* = 9; METH vs saline, respectively, *p* > 0.05; Fig. 3*C*).

As a result, METH treatment did not produce any effect on DGC dendritic morphology with respect to either the dendritic length or on the number of dendritic terminals.

Methamphetamine enhances the enlargement of dendritic spines on both group 2 and group 3 GFP^+^ cells

**Figure 4.**
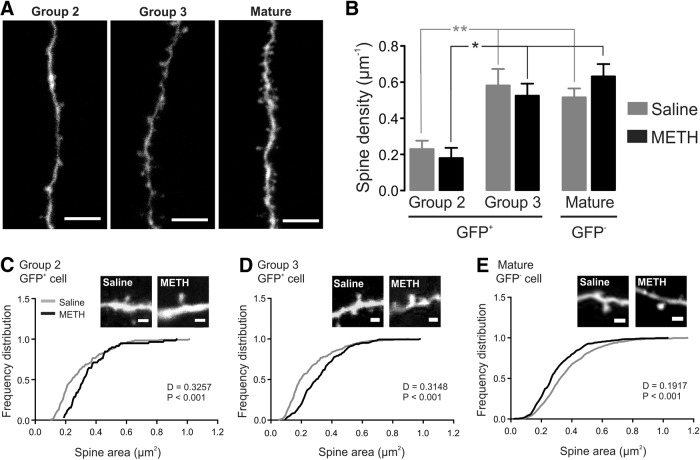
METH effects on spine areas in immature and mature DGCs. ***A***, Representative confocal images of dendritic spines of group 2 and 3 GFP^+^ cells and mature neurons. Scale bar, 5 µm. ***B***, Bar graph depicting spine density in immature and mature DGCs. ***C–E***, Distribution graph of spine area quantified from group 2 (***C***) as well as in group 3 (***D***) GFP^+^ cells and mature DGCs (***E***). *p < 0.05, **p < 0.01, and ***p < 0.001, significantly different using the Kruskal–Wallis test followed by Dunn’s *post hoc* test. Cumulative distributions were compared using Kolmogorov–Smirnov test. Scale bar, 1 µm.

DGC dendritic spines are the major postsynaptic sites of glutamatergic inputs from the entorhinal cortex (Colino and Malenka, 1993). Changes in spine density and morphology are associated with and can contribute to LTP (Ohkawa et al., 2012). We therefore examined whether METH-induced strengthening of LTP was associated with a consistent change in DGC spine morphology. No dendritic spines could be observed in group 1 GFP^+^ cells (data not shown), whereas they could be detected in group 2 and group 3 cells (Fig. 4*A*). Spine density was assessed in GFP^+^ cells in both groups 2 and 3, as well as in mature neurons under saline and METH conditions (Fig. 4*B*). Spine density significantly increased with DGC maturation, reaching a mature level in stage 3 GFP^+^ neurons, as it was not different from that seen in GFP^−^ cells. We found that METH did not induce alterations in spine density in all groups (group 2: 0.18 ± 0.06 vs 0.23 ± 0.05 µm, *n* = 7 vs *n* = 11; Group 3: 0.52 ± 0.07 vs 0.58 + 0.09 µm, *n* = 7 vs *n* = 9; mature: 0.63 + 0.07 vs 0.52 ± 0.05 µm, *n* = 14 vs *n* = 11; METH vs saline, respectively, *p* > 0.05 in all groups; Fig. 4*B*). Interestingly, however, we observed that METH treatment induced an overall significant increase in spine area in GFP^+^ DGCs belonging to group 2 (*n* = 115 in saline and *n* = 59 in METH) and group 3 (*n* = 228 in saline and *n* = 189 in METH; *p* < 0.001; Fig. 4*C*,*D*). This effect was seen only in GFP^+^ differentiating neurons, and not in mature (GFP^−^) DGCs, where a small (albeit significant; *p* < 0.001) reduction was present (*n* = 526 in saline and *n* = 479 in METH; Fig. 4*E*).

Overall, these results suggest that METH treatment affected spine morphology, but not density, in maturing DGCs.

## Discussion

In the present study, we show that repetitive administration of low doses of METH to G42 mice enhanced the differentiation of immature DGCs, induced an enlargement of dendritic spines in differentiating GFP^+^ cells, and facilitated LTP in more mature GFP^+^ as well as in mature DGCs.

We used GFP-expressing neurons in G42 mice, which have been demonstrated to be a useful tool with which to study the functional and anatomical phenotypes of differentiating DGCs (Cabezas et al., 2012; 2013). In this mouse line, GFP expression is transiently expressed in postmitotic DGCs, displaying some aspects of a GABAergic phenotype (Cabezas et al., 2012, 2013). Here we found that METH did not change the total number of GFP neurons, suggesting that METH can promote the differentiation of immature neurons. Indeed, METH decreased the number of GFP^+^ neurons expressing DCX and increased those expressing NeuN without changing the overall fraction of GFP cells coexpressing DCX and NeuN. This could be explained by an acceleration of neuronal maturation. Indeed, the maturation of DG neurons is a dynamic process: DCX^+^ neurons also start expressing NeuN (DCX^+^NeuN^+^) and then stop expressing DCX, expressing NeuN alone (Cabezas et al., 2013). If METH accelerates both these transitions, the intermediate, time-fixed DCX^+^NeuN^+^ phase will be unchanged, because the increased appearance of “early” DCX^+^NeuN^+^ cells balances out the increased disappearance of “late” DCX^+^NeuN^+^ neurons, with a net zero effect. This is supported by the evidence that METH treatment induced a significant decrease of the total fraction of DCX^+^ cells (regardless of whether they also expressed NeuN) and an overall increase of NeuN^+^ cells (regardless of whether they also expressed DCX). Importantly, the METH-induced acceleration of immature neurons is in line with the findings of a previous report (Mandyam et al., 2008) showing the induction of DGC maturation in rats self-administering METH, resulting in an increased population of neurons differentiated from progenitor cells. GFP^+^ cells encompassed a mix of differentiating stages, including an early one (defined as group 1) in which neurons expressed both DCX and NeuN; had limited dendritic branching and spine density; and were characterized by immature firing, high-input resistance, and lack of impinging glutamatergic synaptic transmission. This was likely due to dendrites not reaching the middle molecular layer, and therefore not receiving input from the MPP, indicating that group 1 GFP^+^ cells were not fully integrated into DG circuits yet. We observed that ∼40% of GFP cells expressed only DCX; however, we did not find cells exclusively expressing DCX when we correlated the immunohistochemical phenotype of the GFP^+^ phenotype with their firing. This was probably due to the low number of neurons belonging to group 1 during electrophysiological recordings coupled to immunohistochemistry (*n* = 9 of 36 neurons recorded in total). It is likely that group 1 cells encompass neurons expressing DCX only and DCX and NeuN, representing early maturing neurons. Overall, group 1 neurons likely correspond to an early postmitotic stage (Kempermann et al., 2004; Overstreet-Wadiche and Westbrook, 2006), between 15 and 19 d of age (Zhao et al., 2006; Mongiat et al., 2009; Cabezas et al., 2013). In addition to these early-stage neurons, GFP^+^ cells included NeuN-expressing neurons, which could (group 3) or could not (group 2) fire high-amplitude APs repetitively, and showed increasingly more complex dendritic arborization and higher spine density. Group 2 cells are likely in their third week of age (21–25 d old) as they show reliable EPSCs in response to MPP stimulation (Mongiat et al., 2009), and, in line with other studies (Ge et al., 2007), they showed robust plasticity. Group 3 cells have a mature-like AP firing and longer dendrites that, compared with retroviral-mediated labeling of immature neurons, resemble 28-d-old neurons (Zhao et al., 2006; Mongiat et al., 2009). These neurons responded to TBS with a medium-term (<20 min) potentiation that did not consistently develop into a longer-lasting LTP. This could be due to a different LTP threshold for specific TBS patterns on DGCs at specific developmental stages (Schmidt-Hieber et al., 2007) or differential GluN2B expression during maturation, a process that is necessary for enhanced plasticity (Ge et al., 2007). Moreover, the lack of sustained plasticity might be representative of this class of GFP^+^ cells, which can include a subtype of immature DGC with specific synaptic and/or plasticity features.

We found that METH treatment promoted synaptic plasticity in more differentiated neurons, inducing LTP in group 3 neurons, which could not sustain persistent potentiation in control conditions, and it enhanced the plasticity of mature DGCs. Interestingly, this effect was specific for more differentiated neurons, whereas plasticity in more immature group 2 neurons was unaffected by METH treatment. This could be ascribed to different mechanisms governing the plasticity of DGCs at different developmental stages, of which only the more mature ones may be sensitive to METH. Alternatively, more immature neurons attain an already high plasticity level that could not be potentiated further, corresponding to the peak of their critical period (Ge et al., 2007).

METH-dependent effects on spine areas was more pronounced in DGCs in groups 2 and 3, and had a smaller, opposite effect in GFP^−^ mature neurons, which is in line with previous evidence indicating that LTP promotes spine enlargement in DG immature neurons (Ohkawa et al., 2012).

Overall, however, our results in GFP^+^ differentiating and GFP^−^ mature DGCs suggest that METH-induced effects on LTP might or might not be directly related with those on spine morphology, possibly due to the direct effect on NMDARs, as these receptors have been shown to play a fundamental role in governing plasticity-dependent alterations of spine morphology (Matsuzaki et al., 2004; Zhou et al., 2004; Mahmmoud et al., 2015), and METH treatment was shown to enhance NMDAR mobility (Yamamoto et al., 2006). Alternatively, they might be ascribed to complex interactions between specific DA receptors and NMDARs at different stages of DGC differentiation (Yamamoto et al., 2006; Nai et al., 2010; Yang and Dani, 2014). Future experiments will be required to determine the cellular mechanisms underlying LTP enhancement by METH at specific differentiating stages of DGCs.

Long-term plasticity of hippocampal glutamatergic synapses is believed to be the cellular mechanism of learning and memory (Zeng et al., 2001; Whitlock et al., 2006; Nabavi et al., 2013, 2014; Gruart et al., 2015; Ryan et al., 2015), and it has been shown that a decrease in the plasticity of glutamatergic synapses onto DGCs has a negative effect in memory performance tasks (Farioli-Vecchioli et al., 2008). A specific METH dose and exposure time may induce differential changes at these synapses, affecting both glutamate receptor subunits and glutamate release (Simões et al., 2007; Baptista et al., 2012). It is thus tempting to speculate that the enhanced LTP following METH administration may result in improved memory. In addition to its known recreational use, METH is also prescribed for ADHD treatment, at a usual effective dose of 20–25 mg daily, which is similar to the dose administered to mice in the present study. Importantly, due to its addictive properties, METH is usually prescribed for short periods of time. Individuals in whom ADHD is diagnosed display several aspects of behavior, including hyperactivity, decreased sustained attention, increased behavior variability, and decreased DA transmission (for review, see Himelstein et al., 2000). METH is used to enhance dopaminergic transmission in order to attenuate the symptoms mentioned above, but it may induce dependence, as shown in ADHD animal models (dela Peña et al., 2012).

In conclusion, the present work shows that long-term administration with a low dose of METH promoted the differentiation of immature neurons and enhanced LTP in differentiating and mature DGCs. It will be fundamental to understand how prolonged use of METH will convert such “positive” effects on neurogenesis and synaptic plasticity into the well known dysfunctional effects that strongly impair cognitive performance in METH-addicted subjects.

## Synthesis

The decision was a result of the Reviewing Editor Alfonso Araque and the peer reviewers coming together and discussing their recommendations until a consensus was reached. A fact-based synthesis statement explaining their decision and outlining what is needed to prepare a revision is listed below. The following reviewers agreed to reveal their identity: Clive Bramham, Shaoyu Ge

The reviewers consider that the authors have adequately addressed the criticisms raised about the initially submitted manuscript. There are no further comments.
